# Discriminant Analysis of Traditional Chinese Medicinal Properties Based on Holistic Chemical Profiling by ^1^H-NMR Spectrometry

**DOI:** 10.1155/2020/3141340

**Published:** 2020-03-09

**Authors:** Jie Zhang, Wenna Guo, Qiao Li, Faxin Sun, Xiaomeng Xu, Hui Xu

**Affiliations:** School of Pharmacy, Collaborative Innovation Center of Advanced Drug Delivery System and Biotech Drugs in Universities of Shandong, Key Laboratory of Molecular Pharmacology and Drug Evaluation (Yantai University), Ministry of Education, Yantai University, Yantai, China

## Abstract

Medicinal property, which is closely related to drug chemical profiling, is the essence of traditional Chinese medicine (TCM) theory and has always been the focus of modern Chinese medicine. Based on dozens of classic and commonly used TCM herbs with recognized medicinal properties, the present study just aimed to investigate the feasibility and reliability of medicinal property discriminant by using ^1^H-NMR spectrometry, which provided a mass of spectral data showing holistic chemical profile for multivariate analysis and data mining, including principal component analysis (PCA), Fisher linear discriminant analysis (FLDA), and canonical discriminant analysis (CDA). By using FLDA for two-class recognition, a large majority of test herbs (59/61) were properly discriminated as cold or hot group, and the only two exceptions were Chuanbeimu (Fritillariae Cirrhosae Bulbus) and Rougui (Cinnamomi Cortex), suggesting that medicinal properties interrelate with flavor and body tropism, and all these factors together bring up medicinal property and efficacy. While by performing CDA, 98.4% of the original grouped herbs and 77.0% of the leave-one-out cross-validated grouped cases were correctly classified. The findings demonstrated that discriminant analysis based on holistic chemical profiling data by ^1^H-NMR spectrometry may provide a powerful alternative to have a deeper understanding of TCM medicinal property.

## 1. Introduction

Traditional Chinese Medicine (TCM) has been used to treat and prevent diseases for thousands of years based on a unique theoretical framework [1]. The generalized medicinal property in TCM mainly includes four properties, five flavors, channel tropism, and major function. In a narrower sense, it means four major properties. As firstly described in Shennong Bencaojing, also the Classic of Herbal Medicine, four properties refer to cold (Han), cool (Liang), warm (Wen), and hot (Re) and are usually summed up as cold and hot since the difference between hot and warm, as well as cold and cool, is merely in the extent [2, 3].

In light of TCM theory, the rationale for correct remedy selection is based on relevant syndrome, the biological disorder state or Zheng in Chinese, which is usually classified into cold or hot status showing the imbalance between Yin and Yang in human body [4–6]. Generally speaking, hot ZHENG is caused by excess of Yang, whereas excess of Yin leads to cold ZHENG. Therefore, therapeutic effects mainly depend on medicinal property, as well as the processes they regulate to recover the balance between Yin and Yang [7]. It has become one of the vital TCM treatment principles to cure cold syndrome by medication with hot nature and cure hot syndrome by medication with cold nature, respectively [4, 8]. Resultantly valid medicinal property discriminant is of great significance for prescribing formulary and clinical application of Chinese materia medica.

Medicinal properties represent the types of body reactions after the administration of specific TCM [2, 9]. Traditionally medicinal property is determined according to the curative effects on ZHENG observed in long-term TCM clinical practice. The medicinal herbs with cool or cold property show the efficacy of detoxification, clearing heat, discharging fire, cooling blood, and nourishing Yin and are good at treating hot ZHENG. On the contrary, those with warm or hot property do well in treating cold ZHENG with the efficacy of supporting Yang, warming interior, and dispelling cold. For example, Huangqin, the dry root of *Scutellaria baicalensis* Georgi (Lamiaceae), is a typical cold-property herbal medicine with potent heat-clearing and detoxifying effects, while Fuzi, the prepared branched root of *Aconitum carmichaelii* Debx., exerts strong cold-dispersing efficacy for various cold ZHENG, and is considered as a hot-property medicinal herb. Undoubtedly, the result from such methodology could direct proper application of Chinese medicinal herbs. However, as a highly abstracted part of TCM theories, the medicinal property is far from being deeply understood, and it is difficult to determine the property for all herbs by this way because of large expenditure of money and time. It has become the research focus of Chinese medicine science to explore medicinal property by modern scientific methods for both the worldwide acceptance of TCM and guiding clinical usage of traditional medicinal herbs, as well as discovery and development of new medicinal resources.

With multiple advantages of noninvasion, nondestruction, universal response, high throughput, and good resolution, NMR shows potent metabolic profiling capabilities and has been widely used for metabolomic analysis [10–12]. A recent study shows the role of metabolomics in order to assess the effects of environmental pollution and metabolomics research in the health and safety of marine organisms [13, 14]. At present, there are related researches on aquaculture marine foods. The study of marine biological metabolomics through proton nuclear magnetic resonance can help to better understand its specific physiological functions [15, 16]. Metabolomics is also uses in the field of Chinese medicine; the combination of metabolomic data with multivariate analysis has been used for medicinal property identification. It is highly consistent with the holistic view of TCM and suitable for probing the complex chemical composition of medicinal herb that contains a variety of compounds responsible for efficacy and medicinal property [17, 18].

Based on such a consensus that there are very close connections amongst property, efficacy, and therapeutic material basis of medicinal herb, it may be a viable problem-solving mode to investigate the medicinal property by integrating advanced chemical analysis of holistic composition with data mining techniques. As an emerging high-throughput screening platform, metabolomic analysis can simultaneously profile a wide range of metabolites, thus providing a snapshot of the biological processes that are proximal to a specific phenotype or disease [19]. Nowadays, NMR-based metabolomics that effectively combines NMR assay (mainly ^1^H-NMR) with chemometric methods has become a powerful approach for authenticating and assessing the quality of natural products, characterizing the effects of environmental stressors on organism health as well as tracking the effective components that account for therapeutic effects of Chinese materia medica [20–22]. Herein, we report some interesting results of medicinal property discriminant by using multivariate analysis based on ^1^H-NMR spectrometry providing holistic chemical profiling.

## 2. Materials and Methods

### 2.1. Materials

The herb materials have been enrolled in the Pharmacopoeia of the People's Republic of China (ChP 2015 Edition, Section), including 61 kinds of classic and common-used TCM herbs in the present study. All the herbs were collected from the traditional habitat in China, authenticated, and the voucher specimens were deposited in School of Pharmacy, Yantai University, Yantai, China. According to the records of recognized and definite medicinal property in ChP, 31 cold/cool herbs are listed in Table 1, and the other 30 hot/warm ones in Table 2, respectively. For convenience of data mining and analysis, the herbs with cold medicinal property were further coded as CA, and cool as CB, hot as HA, and warm as HB, respectively. Both Chinese and English denominations, as well as habitat, were listed for each herb.

### 2.2. ^1^H-NMR Assay

Taking into consideration the fact that TCM herbs are traditionally administered as aqueous decoctions, the air-dried medicinal section of each trueborn herb (80∼100 g) was powdered or cut into small pieces and followed by twice extraction with deionized water under reflux (1000 mL, once 1 h). The extracts were combined, evaporated in vacuum to obtain a brown residue, and then freeze-dried for following the NMR assay.

The freeze-dried extract that was equivalent to 200 mg of raw herb material was dissolved in 1 mL DMSO-d6 containing 10% trimethylsilane (TMS) as a reference of chemical shift. After centrifugation (3,000 rpm × 5 min), the supernatant was transferred to a 5 mm o.d. tube for assay, and one-dimensional ^1^H-NMR spectrum was obtained at room temperature and 400.13 MHz proton frequency on a pulse FT NMR spectrometer (Bruker AV-400, Germany); the software package of XWIN-NMR3.5 and *Z*-axis gradients were used. A spectral width of 13966 Hz, an acquisition time of 2.35 s, and a pulse interval of 1.00 s, and the typical acquisition parameters included 65536 data points.

### 2.3. Statistical Analysis

Using the MestReNova NMR processing software (ver. 6.1.1, Mestrelab Research, Santiagode Compostella), the spectrum was processed for phase and baseline correction, and integral calculation by an appropriate interval within the chemical shift (*δ*) ranges from −0.03 to 10.00, among which the chemical shift regions from −0.03 to 0.03 and 2.40 to 2.60 were excluded to eliminate signals of TMS and DMSO. After normalization, ^1^H-NMR spectral data were rearranged to obtain the dataset for further statistical analysis with the rows of data matrix representing the herbs (subjects) and the columns representing chemical shifts (variables), respectively. Principle component analysis (PCA) and discriminant analysis were performed by software of SPSS (ver. 23.0, SPSS, Chicago, IL) and PAST (ver. 1.30, University of Oslo, http://folk.uio.no/ohammer/past).

## 3. Results

### 3.1. ^1^H-NMR Assay

**Figure 1:**
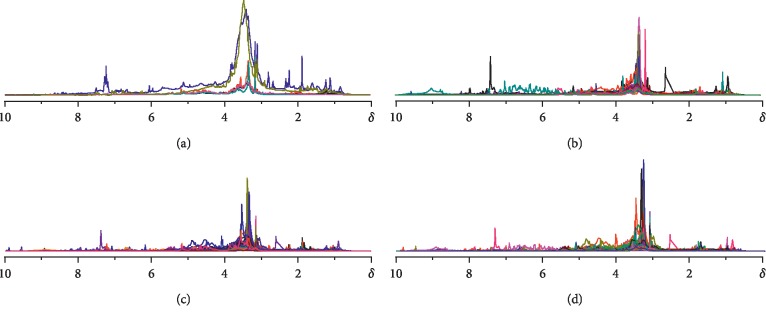
^1^H-NMR spectra of 61 Chinese medicinal herb samples with specific herb nature. (a) Hot; (b) warm; (c) cool; (d) cold (color).

The yield of each herb was calculated as weight percentage of aqueous extract in the raw material of herb, and the results are shown in Tables 1 and 2. As to the normalized ^1^H-NMR spectra illustrated in Figure 1, considerable variation with medicinal property could be found. More concretely, the hot herbs were significantly different from the other three groups, which displayed much lower intragroup variation in both shape and intensity of the peaks at this chemical shift range when compared with other groups; an obvious spectral difference could be even found between hot and warm ones with the former generally showing subsidiary peaks at *δ* 7-8, and the latter at *δ* 1-2, respectively (Figures 1(a) and 1(b)). While for those cold or cool herbs, both intragroup and intergroup comparison displayed relatively high similarity (Figures 1(c) and 1(d)).

### 3.2. Principal Component Analysis (PCA)

**Figure 2:**
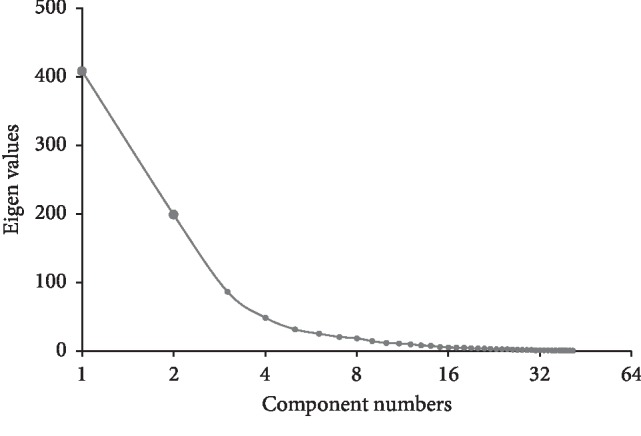
The screen plot of PCA (black-and-white).

**Figure 3:**
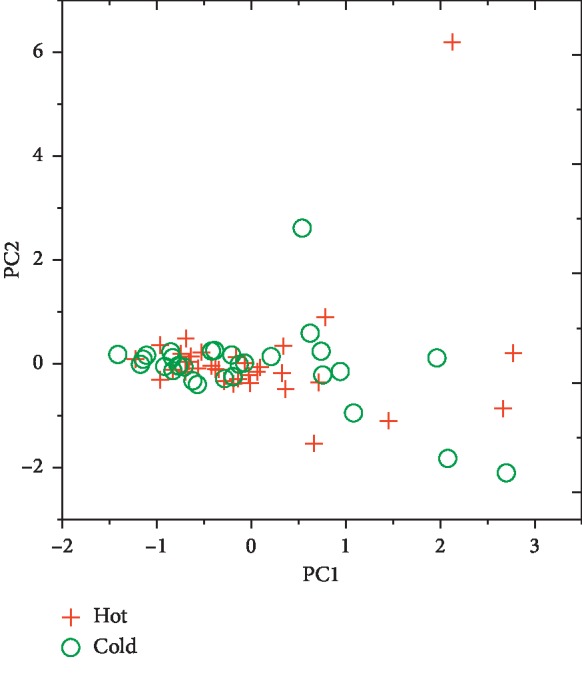
The plot of PCA score (color).

According to the results shown in Figure 2, the first two PCs captured nearly 70% of the total variance, whereas all the other factors displayed much lower eigenvalue with each accounting for less than 8% of the total variance. However, the score plot from PCA based on the ^1^H-NMR spectral dataset of all the herb samples exhibited noticeable intermingling of the black dots representing cold or cool medicinal property and those gray ones representing hot or warm medicinal property (Figure 3).

### 3.3. Fisher Linear Discriminant Analysis (FLDA)

**Figure 4:**
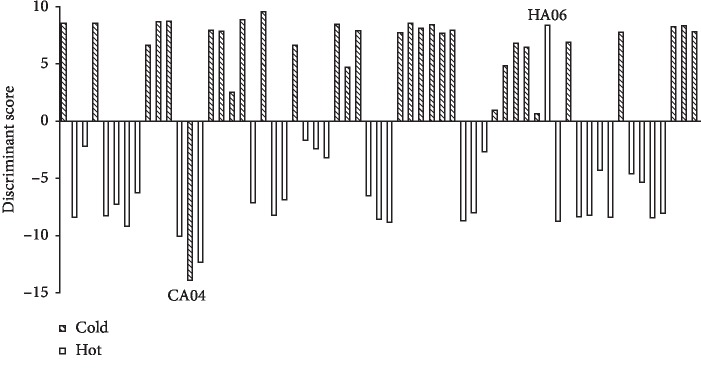
The plot of FLDA score (black-and-white).

FLDA was processed by the software of PAST on the basis of ^1^H-NMR spectral dataset, and all the herbs having plus scores were classified as cold/cool class, while those with minus scores as hot/warm class. As shown in Figure 4, *a* total of 59 herbs were correctly discriminated with the accuracy up to 96.72% (59/61 × 100%). The two exceptions happened to Chuanbeimu (Fritillariae Cirrhosae Bulbus) and Rougui (Cinnamomi Cortex), which were coded as CA04 and HA06, respectively.

### 3.4. Canonical Discriminant Analysis (CDA)

**Figure 5:**
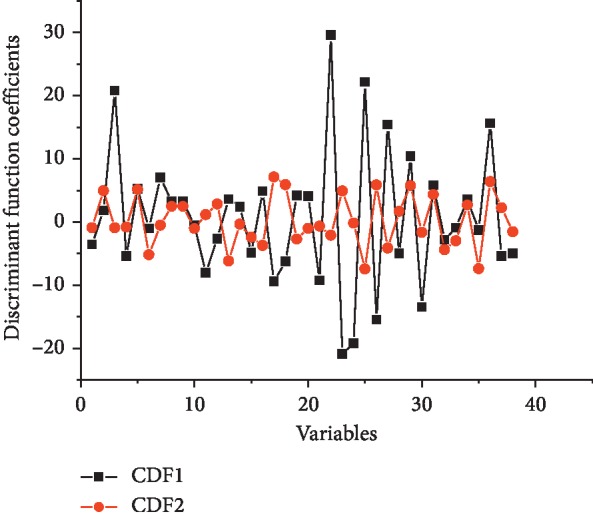
Discriminant function coefficients (color).

As shown in Table 3, the significance test of function coefficients further demonstrated that three CDFs could be built for medicinal property classification with a significance level less than 0.001. Furthermore, the first two CDFs were both indeed linear combination of original variables with canonical correlation coefficients more than 0.9, and they together accounted for more than 90% of the total variance. For the two standardized CDFs, the function coefficients are illustrated in Figure 5, which indicated that the first CDF had a greater contribution than the second one.

**Figure 6:**
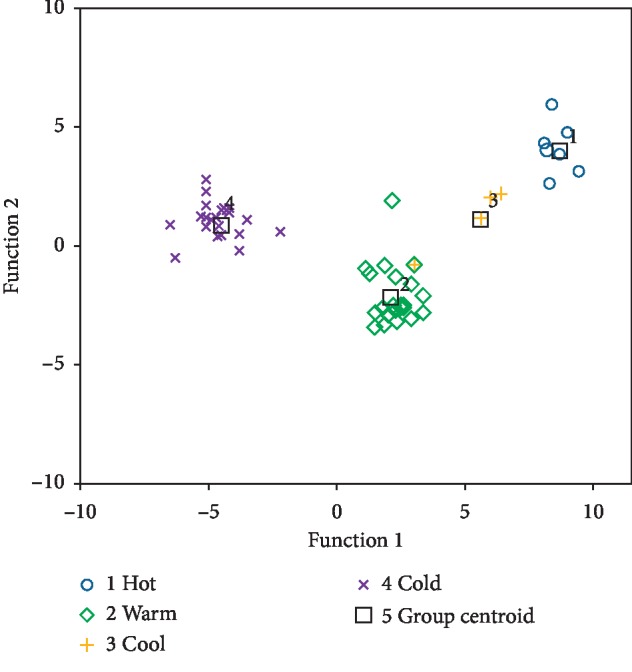
The plot of CDA score (color).

98.4% of the original grouped cases were correctly classified are shown in Table 4, and the only exception was Nvzhenzi (Ligustrl Lucidi Fructus), a traditionally warm herb but classified into cool group by CDA. However, the result in Figure 6 indicates that leave-one-out cross-validated grouped cases that were correctly classified only accounted for 77.0% of the total. As shown in Figure 6, both the red points for the herbs in hot group and the blue ones for those in cold group were fairly centralized and separated from other groups. On the contrary, the other two groups for the herbs with warm (yellow points) or cool (green points) medicinal property displayed obvious commingling with the shortest distance between their group centroids.

## 4. Discussion

The result of ^1^H-NMR spectra indicated that the TCM herbs with different medicinal properties indeed have obvious difference in chemical compositions, and ^1^H-NMR spectroscopic assay may provide an effective way for illustrating and exploring such a variation.

PCA is a kind of unsupervised multivariate statistic approach commonly used for dimensionality reduction and has become a standard technique for data analysis in various fields from neuroscience to computer graphics and process monitoring. The abovementioned spectral datasets from ^1^H-NMR assay of 61 TCM herbs were conducted by PCA. Such findings suggested a poor potency of PCA in discriminating medicinal property of TCM herbs between cold and hot classes.

The Fisher discriminant algorithm has been widely used for pattern recognition through making full use of fault classification information and concentrating all the efforts on finding the optimal Fisher discriminant vector. Using Fisher's discriminant algorithm, linear discriminant analysis (FLDA) for two-class recognition and canonical discriminant analysis (CDA) for multiclass identification were further performed in the present study to improve the poor discrimination of medicinal property classes obtained by the PCA model.

CDA is such a technique that could achieve a fairly convenient description on the relation among various classifications via establishing a small amount of canonical variables for canonical discriminant function (CDF), a linear combination of the original variables. CDA for multiclass discriminant thus was further performed to investigate the four medicinal properties classification of TCM herbs following FLDA in the present study. High recognition efficiency indicated that CDA based on ^1^H-NMR spectral dataset would provide a powerful alternative for the classification of four medicinal properties.

The two exceptions herein from FLDA are Chuanbeimu and Rougui. As of Chuanbeimu, it has sweet flavor to the accompaniment of bitter and cold properties, which belong to the properties of Yang and hence endues some hot/warm property for this medicinal herb. Chuanbeimu is actually one of the vital therapeutic components in various TCM compound formulas for the treatment of thoracic diseases of typhoid due to Yin deficiency, such as Beimu Pills and Yuehua Pills originated from Taiping-Shenghui Fang (the North Song Dynasty, China) and Yinxue Xinwu (the Qing Dynasty, China), respectively. Just because of such dual property of both bitter and sweet flavor, Chuanbeimu is good at treating various heat hyperactivities caused by Yin deficiency. As to Rougui, it may provide a reasonable proof for the interaction between Siqi and SJFC of TCM herbs. According to the discourses in Bencao Xinbian compiled by Shiduo-Chen in the Qing Dynasty, Rougui has a submerging body tropism although it is extremely hot with spicy and sweet flavor, which eventually gives rise to some Yin properties within Yang and the therapeutic effect on lower-Jiao syndromes.

## 5. Conclusion

The findings from the present study indicated that ^1^H-NMR metabolomic approach could be applied to demonstrate the holistic chemical profile of medicinal herb that is closely associated with its medicinal property. Multivariate analysis based on ^1^H-NMR spectral dataset, especially the technique of stepwise CDF for multiclass discriminant, could effectively classify the four medicinal properties, thus providing a feasible means for both the reasonable understanding of the material basis responsible for medicinal property of TCM herbs and discovery and development of new Chinese medicine resource for therapeutic usage. The follow-up research may be concentrated on credibility validation using enlarged-size samples, optimizing the property classification and prediction, as well as assigning the peaks and developing biomarkers.

## Figures and Tables

**Table 1:** Sample information of TCM herbs with cold/cool medicinal property.

Code	Denomination (Chinese/English)	Collection place	Yield (%)
CA01	Cebaiye/Platycladi Cacumen	Liaoning	17.0
CA02	Chaihu/Bupleuri Radix	Henan	17.2
CA03	Cheqianzi/Planttaginis Semen	Jiangxi	10.8
CA04	Chuanbeimu/Fritillariae Cirrhosae Bulbus	Sichuan	14.2
CA05	Dahuang/Rhei Radix Et Rhizoma	Gansu	22.5
CA06	Danzhuye/Lophatheri Herba	Zhejiang	8.8
CA07	Daqingye/Isatidis Folium	Hebei	25.2
CA08	Difuzi/Kochiae Fructus	Zhejiang	16.1
CA09	Dihuang/Rehmanniae Radix	Henan	42.4
CA10	Fangji/Stephaniae Tetrandrae Radix	Zhejiang	11.8
CA11	Gansui/Kansui Radix	Gansu	15.7
CA12	Gualou/Trichosanthis Fructus	Anhui	25.2
CA13	Haizao/Sargassum	Liaoning	12.1
CA14	Hanlian/Ecliptae Herba	Hunan	18.3
CA15	Huangbo/Phellodendri Chinensis Cortex	Sichuan	7.5
CA16	Huanglian/Coptidis Rhizoma	Chongqing	19.0
CA17	Jinyinhua/Lonicerae Japonicae Flos	Shandong	22.1
CA18	Longdan/Gentianae Radix Et Rhizoma	Guizhou	31.8
CA19	Luhui/Aloe	Guangdong	29.0
CA20	Luoshiteng/Trachelospermi Caulis Et Folium	Guangdong	11.9
CA21	Pugongying/Taraxaci Herba	Shandong	26.6
CA22	Qinpi/fraxini Cortex	Liaoning	5.6
CA23	Qumai/Dianthi Herba	Liaoning	12.8
CA24	Tiandong/Asparagi Radix	Yunnan	42.1
CA25	Xixiancao/Siegesbeckiae Herba	Hunan	10.8
CA26	Zhimu/Anemarrhenae Rhizoma	Hebei	9.6
CA27	Zhizi/Gradeniae Fructus	Jiangxi	15.9
CA28	Zicao/Arnebiae Radix	Xinjiang	5.5
CB01	Bohe/Menthae Haplocalycis Herba	Jiangsu	11.2
CB02	Gegen/Puerariae Lobatae Radix	Shandong	46.2
CB03	Nvzhenzi/Ligustri Lucidi Fructus	Shanxi	17.0

**Table 2:** Sample information of TCM herbs with hot/warm medicinal property.

Code	Denomination (Chinese/English)	Collection place	Yield (%)
HA01	Bibo/Piperis Longi Fructus	Hainan	4.2
HA02	Fuzi/Aconiti Lateralis Praeparata	Sichuan	9.9
HA03	Ganjiang/Zingiberis Rhizoma	Sichuan	4.6
HA04	Gaoliangjiang/Alpiniae Officinarum Rhizoma	Guangdong	7.4
HA05	Hujiao/Piperis Fructus	Hainan	1.4
HA06	Rougui/Cinnamomi Cortex	Guangxi	6.2
HA07	Wuzhuyu/Euodiae Fructus	Guizhou	17.2
HA08	Xianmao/Curculiginis Rhizoma	Sichuan	14.2
HB01	Baijiezi/Sinapis Semen	Anhui	12.7
HB02	Buguzhi/Psoraleae Fructus	Sichuan	15.6
HB03	Cangzhu/Atractylodis Rhizoma	Liaoning	33.4
HB04	Caodoukou/Alpiniae Katsumadai Semen	Guangdong	7.0
HB05	Chenpi/Citri Reticulatae Pericarpium	Guangdong	31.6
HB06	Chuanxiong/Chuanxiong Rhizoma	Sichuan	25.4
HB07	Duzhong/Eucommiae Cortex	Sichuan	8.1
HB08	Fabanxia/Pinelliae Rhizoma Praeparatum	Sichuan	13.6
HB09	Gansong/Nardostachyos Radix Et Rhizoma	Sichuan	11.9
HB10	Gaoben/Ligustici Rhizoma Et Radix	Sichuan	17.1
HB11	Honghua/Carthami Flos	Xinjiang	31.0
HB12	Houpu/Magnoliae Officinalis Cortex	Sichuan	9.8
HB13	Mahuang/Ephendrae Herba	Liaoning	12.6
HB14	Mugua/Chaenomelis Fructus	Yunnan	27.5
HB15	Muxiang/Aucklandiae Radix	Yunnan	40.6
HB16	Qianghuo/Notopterygii Rhizoma et Radix	Gansu	29.4
HB17	Tanxiang/Santali Albi lignum	Guangdong	7.3
HB18	Tiannanxing/Arisaematis Rhizoma	Liaoning	14.5
HB19	Weilingxian/Clematidis Radix Et Rhizoma	Liaoning	25.4
HB20	Xixin/Asari Radix et Rhizoma	Liaoning	11.8
HB21	Yanhusuo/Corydalis Rhizoma	Zhejiang	13.2
HB22	Yinyanghuo/Epimedii Folium	Shanxi	14.6

**Table 3:** Significance testing of CDFs obtained by the stepwise method.

CDF	1	2	3
Eigenvalue	11.761	6.407	1.644
Percentage of variance	59.4	32.3	8.3
Canonical correlation	0.960	0.930	0.789
Test of function(s)	1 through 3	2 through 3	3
Wilk's lambda	0.004	0.051	0.378
Chi-square	212.564	114.529	37.433
Sig.	0.000	0.003	0.449

**Table 4:** Classification results of CDA.

Original group	Total	Counts of mistakes	Mistake group
Hot	6	0	—
Warm	24	1	Cool
Cool	6	0	—
Cold	25	0	—

## Data Availability

The data including tables and figures used to support the findings of this study are included within the article.
